# Tumor microbial biodiversity and microsatellite instability in colorectal cancer

**DOI:** 10.1371/journal.pone.0359555

**Published:** 2026-09-25

**Authors:** Calen Mendall, Meredith A. J. Hullar, Keith R. Curtis, Courtney M. Hill, Claire E. Thomas, Ningxin Ma, Timothy W. Randolph, Rachel C. Malen, Adriana M. Reedy, Scott LaBrie, John D. Potter, Shuji Ogino, Polly A. Newcomb, Amanda I. Phipps

**Affiliations:** 1 Department of Epidemiology, School of Public Health, University of Washington, Seattle, Washington, United States of America; 2 Public Health Sciences Division, Fred Hutchinson Cancer Center, Seattle, Washington, United States of America; 3 Clinical Research Division, Fred Hutchinson Cancer Center, Seattle, Washington, United States of America; 4 Centre for Public Health Research, Massey University, Wellington, New Zealand; 5 Department of Epidemiology, Harvard T.H. Chan School of Public Health, Boston, Massachusetts, United States of America; 6 Department of Pathology, Brigham and Women’s Hospital and Harvard Medical School, Boston, Massachusetts, United States of America; 7 Broad Institute of MIT and Harvard, Cambridge, Massachusetts, United States of America; University of the Pacific, UNITED STATES OF AMERICA

## Abstract

**Background:**

Growing evidence links the gut microbiome to colorectal cancer (CRC) progression, with certain bacterial species enriched in specific molecular tumor subtypes. DNA mismatch repair deficiency in CRC, evidenced by the presence of microsatellite instability (MSI), has been consistently associated with a favorable prognosis, and may be related to certain aspects of the microbiome. Here, we examined the relationship between tumor microbial biodiversity and MSI status.

**Methods:**

Diagnostic tumor tissue samples were obtained from the Seattle site of the Colon Cancer Family Registry (SCCFR) and a companion study; both recruited patients diagnosed with incident CRC from 1998 to 2007. MSI status assessment and prokaryotic *16S rRNA* gene sequencing was performed on the tumor tissue samples. We used an adaptive test of alpha-diversity (aMiAD) to estimate the association between microbial biodiversity and MSI status. We performed differential abundance analysis with ANCOM-BC to identify enriched genera in tumor tissue, based on dichotomized MSI status. Analyses were adjusted for age, sex, smoking history, and tumor location (N = 632).

**Results:**

The adaptive aMiAD effect estimate was −1.08 (p = 0.29), suggesting that MSI-high tumors had lower estimated alpha-diversity, though this difference was not statistically significant. We identified 20 differentially abundant genera in CRC tumors according to MSI status, with 8 enriched genera and 12 depleted genera in MSI-high tumors. The most strongly enriched genera in MSI-high tumors were *Gemella* and *Lawsonella*, while *Sporolactobacillaceae* and *Cloacibacterium* were the most strongly depleted*. Fusobacterium* was enriched in MSI-high tumors only after subsetting the genus to *Fusobacterium nucleatum* specific sequences.

**Conclusions:**

We did not detect a statistically significant association between the adaptive alpha-diversity measure and MSI status, though individual measures concordantly estimated a depletion of alpha-diversity in the MSI-high tumors. We found evidence of differential abundance of certain genera dependent on MSI status, including several novel associations.

## Introduction

Colorectal cancer (CRC) is a heterogeneous disease with several well-characterized molecular phenotypes and subtypes, which have been suggested to vary in their etiology and to exhibit differing clinical features and prognoses [1]. Microsatellite instability (MSI) is a marker of the mismatch repair (MMR) deficient CRC phenotype, where deleterious mutations or epigenetic silencing of MMR genes results in non-functional or unexpressed proteins responsible for correcting DNA replication errors [1,2]. High levels of MSI (MSI-high) in a tumor are present in an estimated 12–17% of CRC cases [2], and are generally associated with more favorable prognosis [1,2]. MMR deficiency appears to occur without germline mutations in MMR genes in 9–14% of CRC cases [2], making it important to understand the factors leading to sporadic MMR-deficient CRC.

Dysbiosis, characterized by disruption to the composition and balance of taxa within a healthy colorectal tissue-associated microbiome, is believed to have a causal role in CRC pathogenesis [3,4]. At the genera level, *Bacteroides, Escherichia, Fusobacterium,* and *Salmonella* are associated with and enriched in CRC tumor tissue [5]. Furthermore, several bacterial species are enriched in CRC tumor tissue [5–7]; *Fusobacterium nucleatum* (*F. nucleatum*), in particular, is strongly associated with CRC, where it is suspected to induce inflammation in colorectal tissue [3,8]. Inflammation of colorectal tissue appears to play an important role in the development of CRC [2]. Numerous studies have identified strong associations between *F. nucleatum* colonization and CRC [8,9], and between *F. nucleatum* and the MMR-deficient (MSI-high) phenotype [10,11]. *F. nucleatum* colonization is associated with a worse CRC prognosis [11–13], whereas the MSI-high phenotype is generally associated with a favorable prognosis [1,2]; thus, understanding the factors that contribute to this contrast remains important.

Although certain individual bacterial species are likely to play a role in shaping CRC phenotype and prognosis, aspects of the overall composition of the microbiome in CRC may also be relevant. In particular, associations between microbial biodiversity and MSI-high CRC have been identified previously: both higher biodiversity in MMR-deficient (MSI-high) CRC tumors than in MMR-proficient (microsatellite stable, henceforth MSS/MSI-low, reflecting intact MMR systems) tumors and genera-specific shifts in composition have been found [14–17]. The generalizability of these results remains uncertain because the estimated effect depended on the choice of alpha-diversity metric and not all previous works adjusted for important confounders, such as tumor location and smoking history [14–16]. There are a number of commonly used alpha-diversity metrics that prioritize different taxa based on relative abundance in the community and/or phylogenetic relationships between taxa. Without *a priori* reasons to select one measure over others, it is common practice to use multiple measures; however, it is challenging to interpret results when the measures disagree. Adaptive tests help resolve this by providing valid tests and effect estimates that compare a set of pre-specified alpha-diversity metrics and maintain control of type I error rates [18].

Here, we examined the association between tumor alpha-diversity and MSI in CRC using an adaptive test of alpha-diversity (adaptive microbiome α-diversity-based association analysis – aMiAD) that incorporates both abundance-based and phylogeny-based alpha-diversity metrics [18], and adjusted for important confounders: age, sex, tumor location, smoking history. We also explored whether *F. nucleatum* positivity in tumor tissue affected the presence of an association between alpha-diversity and MSI in CRC. Finally, we searched for differentially abundant genera in MSI-high tumors compared to MSS or MSI-low tumors.

## Methods

### Study population

Study data come from the Seattle site of the Colon Cancer Family Registry (SCCFR) and a smaller, parallel study of CRC [19,20]. Both studies conducted population-based recruitment of participants with incident diagnoses of invasive CRC, identified through the Seattle-Puget Sound site of the Surveillance, Epidemiology, and End Results (SEER) cancer registry network. Recruitment was performed in two phases; in Phase I, recruited CRC cases resided in select Washington counties and were aged 20–74 years old at diagnosis, with diagnosis dates between January 1998 and June 2002. In Phase II, recruited incident CRC cases were all younger than 50 years old at the time of diagnosis, were from an extended set of Washington counties, and were diagnosed between April 2002 and July 2007. All participants gave written informed consent for the collection and analysis of their biospecimens and medical reports, and provided verbal consent via trained interviewers for use of information collected through questionnaire responses. The Institutional Review Board at the Fred Hutchinson Cancer Center approved this study. De-identified data were accessed between February 15, 2025 and June 8, 2025.

### Biospecimen and data collection

Participants completed a questionnaire at enrollment that collected basic demographic information, family history of cancer, limited aspects of diet, and potential CRC risk factors (N = 2,318). Biospecimens were collected from diagnostic tumor tissue and were stored as formalin-fixed paraffin-embedded (FFPE) blocks or sections on slides. Tissue samples were assessed for several molecular markers, including MSI status, somatic mutations in *KRAS* and *BRAF*, and the CpG island methylator phenotype (CIMP), as described elsewhere [21]. For a subset of participants (N = 902), characterization of the tumor-associated microbiome was also conducted, including sequencing of the bacterial *16S rRNA* gene on a further subset (N = 680), targeting the V4 hypervariable region, using microbial DNA extracted from tumor samples with a modified extraction protocol for FFPE samples as previously described [13,22]. Additionally, targeted droplet digital polymerase chain reaction (ddPCR) of the *F. nucleatum* specific homolog of the universally conserved transcription elongation factor N-utilization substance G (*nusG*) gene was used to detect and quantify this species in the tumor tissue [22].

### *16S rRNA* sequencing and pre-processing

*16S rRNA* gene sequencing was performed at the Molecular Research LP (Shallowater, TX) using paired-end reads on the Miseq platform (Illumina); sequence pre-processing was performed at the Fred Hutchinson Cancer Center. In brief, the sequencing pre-processing was performed with USEARCH-UNOISE3 [23,24] (usearch v11.0.667_i86linux64) and QIIME 2 [25] to generate amplicon sequencing variants (ASVs). We used an ASV abundance threshold of 0.001% to remove pipeline-specific ASVs similar to 0.002% used in Prodan et al. [26]. ASVs were taxonomically classified using the classify-sklearn module with SILVA 138 taxonomy [27]. An initial phylogeny was generated in QIIME 2 with MAFFT [28] for multiple sequence alignment and fasttree2 [29] for phylogeny construction. Sample contamination was addressed with SCRuB [30] (v0.0.1) and a final ASV filter was applied at the genus level (>0.0009 abundance). Additional genus-level filtering was conducted based on both passing LOD and LOQ levels (or just LOD levels if the genera was previously observed in CRC tumors) [31,32], as well as by excluding probable contaminants [32–34], leaving a total of 48 genera in the final analysis. 1403 ASVs passed filtering and were kept in the generation of alpha-diversity measures and the final phylogeny used in this study. To generate the phylogeny-based alpha-diversity metrics, prepared *16S rRNA* gene sequences were aligned with the Super5 algorithm using MUSCLE [35] (v5.3.osxarm64). A phylogeny was generated from aligned sequences using VeryFastTree [36] (v4.0.4). A representative *16S rRNA* gene sequence from the gut archaea *Methanobrevibacter intestini* (RefSeq: PQ_670969.2) [37] was included in the alignment and clustering steps to root the phylogeny.

### Alpha diversity

Tumor-specific bacterial alpha-diversity was measured from the tissue samples of CRC tumors using the processed *16S rRNA* gene sequencing. The primary analysis was performed using an adaptive measure of diversity (aMiAD) – which incorporates six measures of alpha-diversity (S1 Table): observed Richness [38], the Shannon index [39], the Simpson index [40], Phylogenetic Diversity (PD) [41], Phylogenetic Entropy (PE) [42], and Phylogenetic Quadratic Entropy (PQE) [43] – to estimate the magnitude and direction of association between alpha-diversity and MSI in tumors. Different taxa are prioritized depending on selection of the alpha-diversity metric: the Simpson index up-weights the most relatively abundant taxa, the Shannon index moderately weights each taxa by their relative abundance, and Richness gives equal weight to all taxa [18]. PD, PE, and PQE are analogous to Richness, the Shannon index, and the Simpson index, respectively, and incorporate the relatedness of taxa using phylogenetic branch lengths in addition to relative abundances, where more distantly related taxa receive higher weights [18,42]. Phyloseq [44] (v1.50.0) and Enteropart [45] (v1.6-16) were used to calculate the individual non-phylogenetic and phylogenetic alpha-diversity metrics, respectively.

### MSI status

MSI status in CRC tumors was dichotomized as MSS or MSI-low versus MSI-high, based on either the proportion of unstable loci from a targeted sequencing panel of 10 markers or on immunohistochemical (IHC) staining of four MMR proteins (MLH1, MSH2, MSH6, and PMS2), as previously described [19].

### *F. nucleatum* positivity

Characterization of tumor *F. nucleatum* positivity was assessed by ddPCR of the *F. nucleatum nusG* gene [22]. To be classified as *F. nucleatum* positive, a sample needed to have at least 4.1 copies/10 ng tissue of *nusG* present to exceed the limit of quantitation (LOQ) for the assay. For analyses examining *F. nucleatum* as an effect modifier of the relationship between microbial biodiversity and MSI-high CRC, *F. nucleatum* positivity in tumor was treated as a binary measure (present, absent) and was paired with the Shannon index, as the Shannon index offers a balanced weighting of relative abundance compared to Richness and the Simpson index [18].

### Confounders

Several measures were controlled for as important confounders of the relationship between alpha-diversity and MSI status in CRC: age at diagnosis, self-reported sex, tumor location, and tobacco smoking status at CRC diagnosis. Confounders were selected based on described associations with both the gut microbiome and the MSI phenotype [1,46–52], and where they were not expected to be on the assumed causal pathway between alpha-diversity and MSI status. There are other likely confounders, such as alcohol consumption, dietary habits, and race, that were not included in analyses due to variability in reporting and small sample sizes. Tumor location was classified based on the assigned ICD-O-3 codes as proximal (C18.0, C18.2, C18.3, and C18.4), distal (C18.6, C18.7, and C18.5), or rectal (C19 and C20) [53]. Smoking history was classified based on a participant’s self-reported smoking history into three categories (never, former, or current smoker) [47].

### Statistical analysis

All participants needed complete classification of MSI status and to have passed *16S rRNA* gene sequencing quality control for inclusion in the analysis (N = 632). For analyses involving a participant’s tumor *F. nucleatum* positivity status, successful classification of *F. nucleatum* positivity status by targeted ddPCR of the *nusG* gene was also required (N = 627). All analyses were performed in R [54] (version 4.4.0) unless otherwise specified.

To measure the association between alpha-diversity and MSI, we applied the aMiAD method [18] (v.2.0) using multiple logistic regression of the six alpha-diversity measures on dichotomous MSI status. The aMiAD methodology estimates standardized effect scores (MiDivES) and p-values for each individual alpha-diversity measure using a residual permutation method to generate null models [18]. The measure with the strongest support is selected for estimating the adaptive effect score; to control type I error rates, the adaptive microbial diversity effect score (aMiDivES) and related p-value are re-estimated using a further set of permutations. This approach yields valid estimates of both the effect direction and magnitude of the individual alpha-diversity measures and for the adaptive measure. We used 5000 permutations to estimate the test statistics for this measure on complete data.

To evaluate whether the relationship between tumor alpha-diversity and MSI status was modified by *F. nucleatum* positivity, we fit adjusted logistic regression models using the Shannon index in relation to MSI status; these models also included tumor *F. nucleatum* positivity with and without an interaction term between alpha-diversity and tumor *F. nucleatum* positivity. A Wald test [55] was used to test whether the interaction term was statistically significant. To preserve power and reduce bias in these models, missing covariate values were multiply imputed using the expectation-maximization with bootstrapping algorithm from the Amelia package [56] (v1.8.3). We generated 10 imputed datasets, imputing values only for age at diagnosis, sex, smoking status, and tumor location. Regressions were run independently on each imputed dataset, and results were then pooled with the Mice package [57] (v3.17.0) using the mean coefficient value for final point estimates and Rubin’s rules [58] to estimate variance.

We used Analysis of Compositions of Microbiomes with Bias Correction (ANCOM-BC, v2.8.1) [59] to assess differential abundance in genera between MSI-high and MSI-low/MSS tumors, and adjusted for the discussed confounders. The ANCOM-BC methodology offers strong control of the false discovery rate (FDR), allows for control over the inclusion of structural zeros in the analysis, and provides sensitivity analyses to determine whether results are robust to pseudocount variation [59,60]. Multiple comparison correction was performed using the Benjamini-Hochberg procedure [61]. We used a prevalence cut-off of 0.05 for inclusion in the analysis and allowed structural zeros to be included using both identification criteria described by the authors [59]. With this prevalence cut-off, only 45 genera were considered in the analysis.

### Sensitivity analyses

To further examine how specific alpha-diversity metrics were associated with MSI status, we fit logistic regression models on the multiple imputed data and individually modelled each alpha-diversity metric on MSI status adjusted for potential confounders. To allow for more direct comparisons of individual alpha-diversity metrics on a common scale [62–64], the alpha-diversity metrics were transformed to effective number of species (ENS) values and fitted in adjusted logistic regression models. The phylogeny-based diversity metrics were transformed to ENS values with a standardized phylogeny with height of 1 (agnostic to the true scale of the phylogeny). An ENS value corresponds to the estimated number of equally distantly related taxa of equal abundance that would have generated the same alpha-diversity value as was observed in the data for that measure [62–64]. We also re-conducted the aMiAD analysis with these transformed ENS values. To further explore effect modification by *F. nucleatum* on the relationship between alpha-diversity and MSI status, we fit additional logistic regression models of the Shannon index on MSI status; these models were fit separately on the subset of individuals with *F. nucleatum* positive tumors and the subset of individuals with *F. nucleatum* negative tumors. ANCOM-BC analysis was repeated at the family level using the same parameters as the main analysis. As *F. nucleatum* has a well-described enrichment in MSI-high tumor tissues [8,10,11] and some of the *Fusobacterium* ASVs were annotated as a species other than *F. nucleatum*, we ran the ANCOM-BC analysis on *F. nucleatum* specific ASVs. We used BLASTn [65,66] (Command line v2.16.0, megablast; word size 28, using the 16S_ribosomal_RNA database) to identify and subset *Fusobacterium* ASVs to ASVs with the top hit matching one of the 4 present *F. nucleatum* subspecies: *F. nucleatum nucleatum, F. animalis, F. polymorphum,* and *F. vincentii* [67]. Finally, we conducted sex-stratified aMiAD and genus level ANCOM-BC analyses since there is strong support for associations between sex, MSI status, and the gut microbiome [50,51,68,69], with additional work indicating that sex modifies the strength of the alpha-diversity and CRC relationship [70].

## Results

Of the 632 participants who met inclusion criteria, 125 (19.8%) had MSI-high tumors. The overall cohort was approximately evenly distributed by sex (52% female), though the MSI-high group had a higher proportion of female participants (67%) than the MSS/MSI-low group (49%) (Table 1). The majority of participants were White (79%). Proximal tumors were the most frequently observed overall (46%) and the proportion of participants with proximal tumors differed markedly by MSI status (86% of MSI-high participants vs. 36% of MSS/MSI-low participants). Most participants were diagnosed with regional (stage II-III) CRC (58%), while 12% were diagnosed with distant (stage IV) disease. Participants with MSI-high tumors were more likely to be current smokers at CRC diagnosis (14% vs. 10% in those with MSS/MSI-low tumors and 11% overall). The prevalence of *F. nucleatum* positivity was markedly different by MSI status, with 46% vs. 17% of cases with MSI-high vs. MSI-low/MSS tumors, respectively, exhibiting *F. nucleatum* positivity. Overall, Shannon diversity was slightly higher in participants with MSI-low/MSS CRC compared to individuals with MSI-high CRC, with medians of 2.13 and 1.98, respectively (S2 Table).

**Table 1:** Summary of study participant characteristics stratified by MSI status and overall.

Characteristic	MSI-high N = 125^*1*^	MSS/MSI-low N = 507^*1*^	Overall N = 632^*1*^
**Sex**			
Female	84 (67%)	246 (49%)	330 (52%)
Male	41 (33%)	261 (51%)	302 (48%)
**Age**	63 (47, 69)	59 (49, 67)	60 (48, 67)
**Race** ^***2***^			
White	90 (72%)	384 (76%)	474 (75%)
More Than One Race	20 (16%)	57 (11%)	77 (12%)
Other	5 (4.0%)	42 (8.3%)	47 (7.4%)
Unknown	10 (8.0%)	24 (4.7%)	34 (5.4%)
**Tumor site**			
Proximal	107 (86%)	183 (36%)	290 (46%)
Distal	12 (9.6%)	157 (31%)	169 (27%)
Rectal	3 (2.4%)	157 (31%)	160 (25%)
Unknown	3 (2.4%)	10 (2.0%)	13 (2.1%)
***F. nucleatum* status**			
Positive	56 (45%)	88 (17%)	144 (23%)
Negative, or below LOQ	67 (54%)	416 (82%)	483 (76%)
Unknown	2 (1.6%)	3 (0.6%)	5 (0.8%)
**Smoking history**			
Current	18 (14%)	52 (10%)	70 (11%)
Former	58 (46%)	236 (47%)	294 (47%)
Never	49 (39%)	219 (43%)	268 (42%)
**Stage**			
Local	45 (36%)	143 (28%)	188 (30%)
Regional	73 (58%)	283 (56%)	356 (56%)
Distant	5 (4.0%)	67 (13%)	72 (11%)
Unknown	2 (1.6%)	14 (2.8%)	16 (2.5%)

^*1*^n (%); Median (Q1, Q3).

^*2*^Stratified numbers for other self-reported racial categories are censored due to small numbers. The overall analytic population included N = 22 participants who self-reported Black or African American race, N = 18 who self-reported Asian race, N < 10 who self-reported their race as Native Hawaiian or Other Pacific Islander, and N < 5 who self-reported American Indian or Alaska Native as their racial identity.

**Fig 1:**
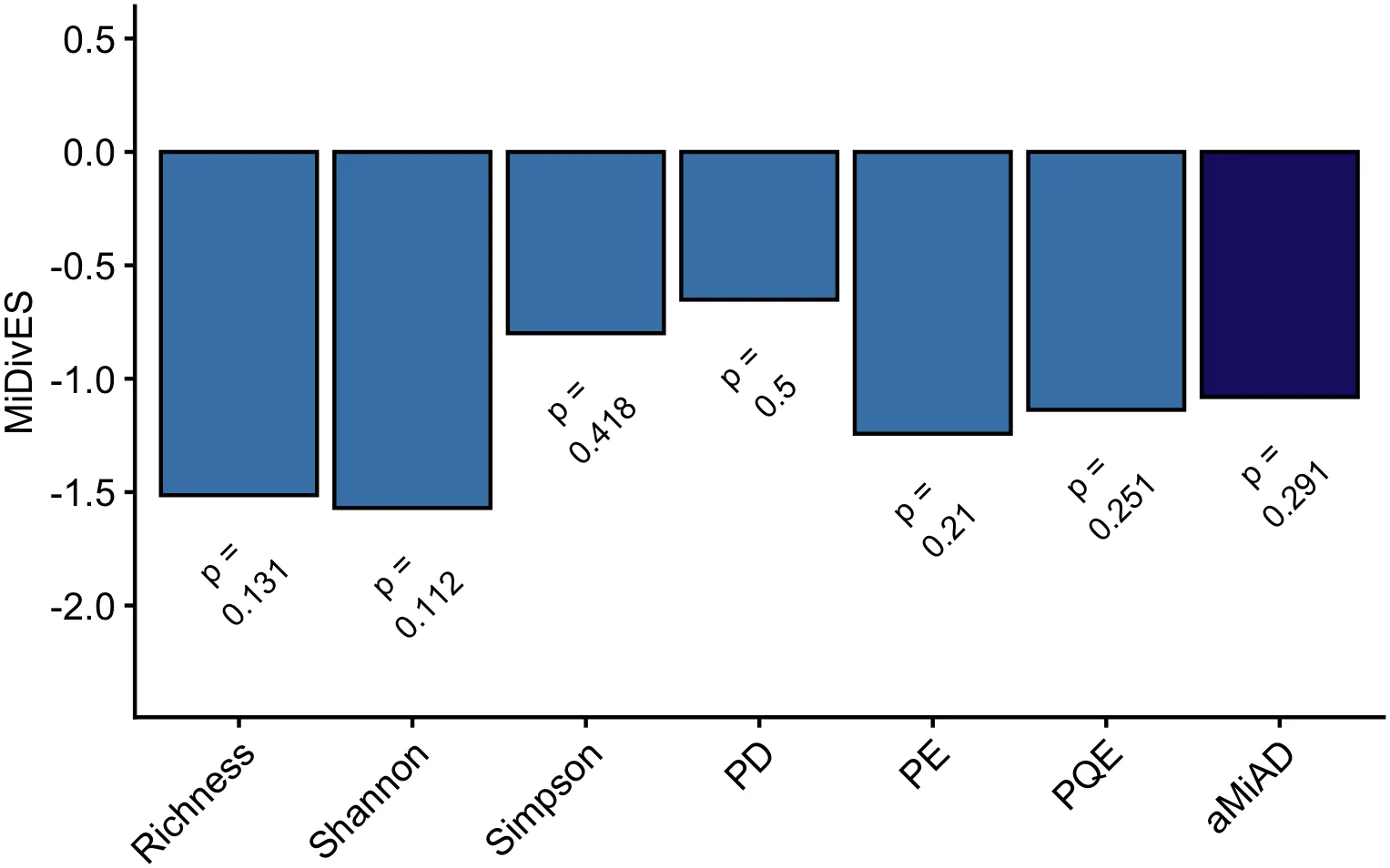
Adaptive and individual measures of alpha-diversity show inverse relationships with MSI status. Individual effect score estimates (MiDivES: light blue) from aMiAD analysis of the 6 alpha-diversity metrics and the effect estimate of the adaptive measure aMiAD (aMiDvES: dark blue). Analysis adjusted for age at diagnosis, sex, smoking history, sex, and tumor location. Effect score estimates below 0 represent negative associations of alpha-diversity with MSI status, with the magnitude of the result indicating the relative strengths of the associations. Abbreviations: PD; Phylogenetic Diversity; PE, Phylogenetic Entropy; PQE, Phylogenetic Quadratic Entropy; MiDivES, Microbial Diversity Effect Score; aMiAD, adaptive microbiome alpha-diversity-based association analysis.

All aMiAD effect scores of alpha-diversity showed an inverse association with MSI status, where lower alpha-diversity was associated with higher odds of having MSI-high CRC after adjusting for sex, age at diagnosis, smoking history, and tumor location (Fig 1). The magnitude of these associations was the greatest for Richness and Shannon diversity (MiDivES −1.51 and −1.57, respectively). The adaptive effect score, aMiDivES, was −1.08; however, there was no evidence of a statistically significant association between any of the individual alpha-diversity measures nor the adaptive aMiAD effect score and MSI status (aMiAD p = 0.295) (Fig 1). Similarly, sensitivity analyses of individual models for each of the alpha-diversity measures on the ENS scale and as nominal alpha-diversity values showed inverse associations with MSI status across all measures, though no individual alpha-diversity measures were statistically significant on either scale (S1 Fig). All aMiAD effect scores remained negative when fit with the alpha-diversity measures on the ENS scale but, notably, the strength of the associations increased for all individual measures that were functionally different (i.e., richness and PD did not change on ENS scale as parameterized here). In particular, the Simpson index increased in magnitude to an effect score of −2.0 from −0.8 (S2 Fig). In sex-stratified analyses on the ENS scale, the aMiAD score was similar between men (−1.06, p = 0.33) and women (−1.19, p = 0.251), however, PE and PQE effect estimates varied greatly by sex (S3 Fig).

**Fig 2:**
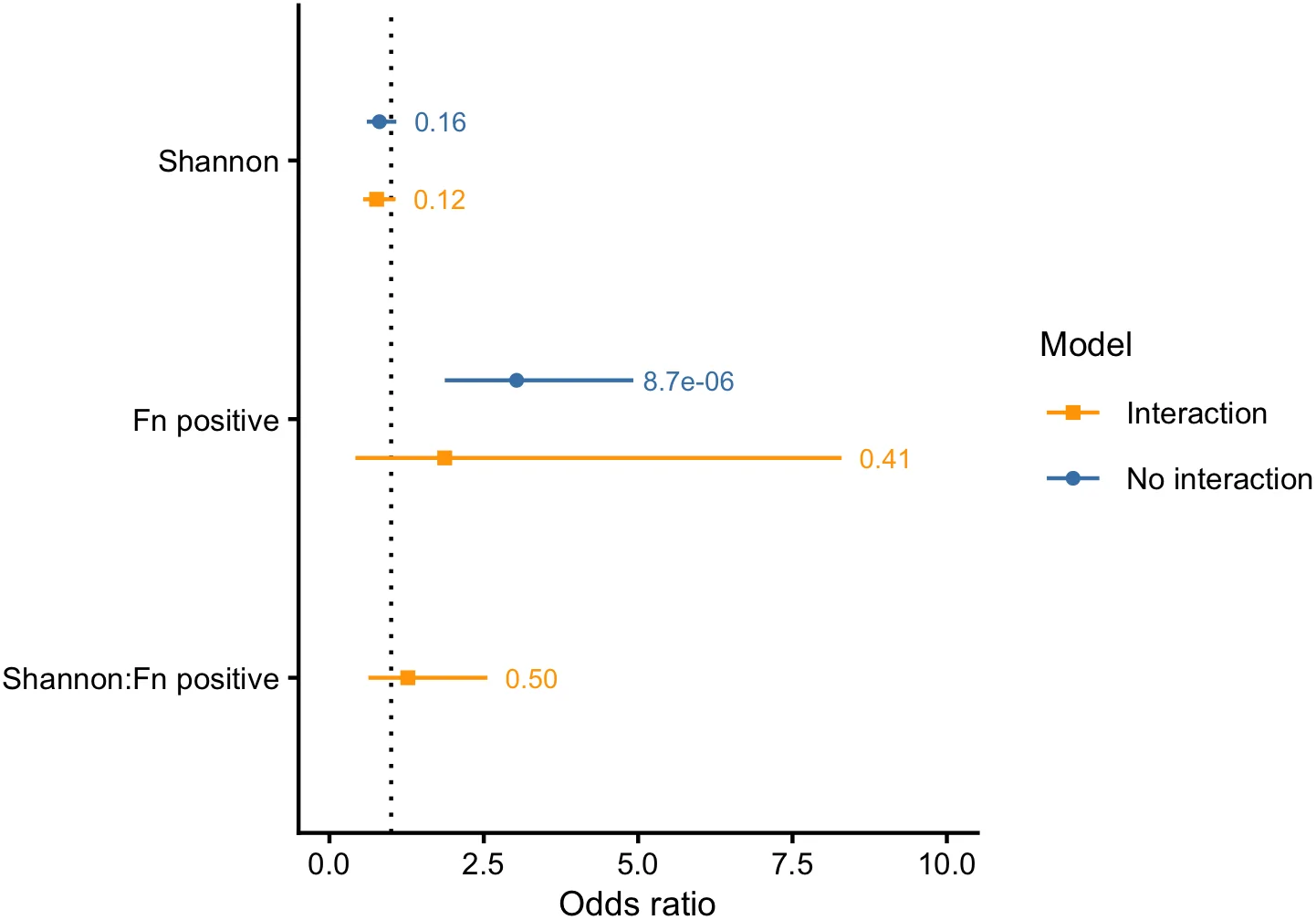
*F. nucleatum* (Fn) positivity is positively associated with MSI-high status. Forest plots of estimated odds ratios of the Shannon index and *F. nucleatum* positivity from ddPCR on MSI status from models with and without interaction between the two measures. Both models were otherwise adjusted for age at diagnosis, sex, smoking history, sex, and tumor location. P-values displayed next to their respective estimate.

In the model of the Shannon index on MSI status that included a tumor *F. nucleatum* positivity term, we found a strong association between *F. nucleatum* positivity and MSI status, with *F. nucleatum* positive tumors estimated to have 3.03 (95% CI 1.87–4.92) times the odds of MSI-high status compared to *F. nucleatum* negative tumors (Fig 2). However, we found no evidence for statistically significant effect modification by *F. nucleatum* positivity on the association between the Shannon index on MSI status (p = 0.504) (Fig 2). Sensitivity analysis of models fit on participant subsets by *F. nucleatum* positivity showed an attenuated estimate of the association between Shannon index and MSI status in *F. nucleatum* positive tumors (S4 Fig).

**Fig 3:**
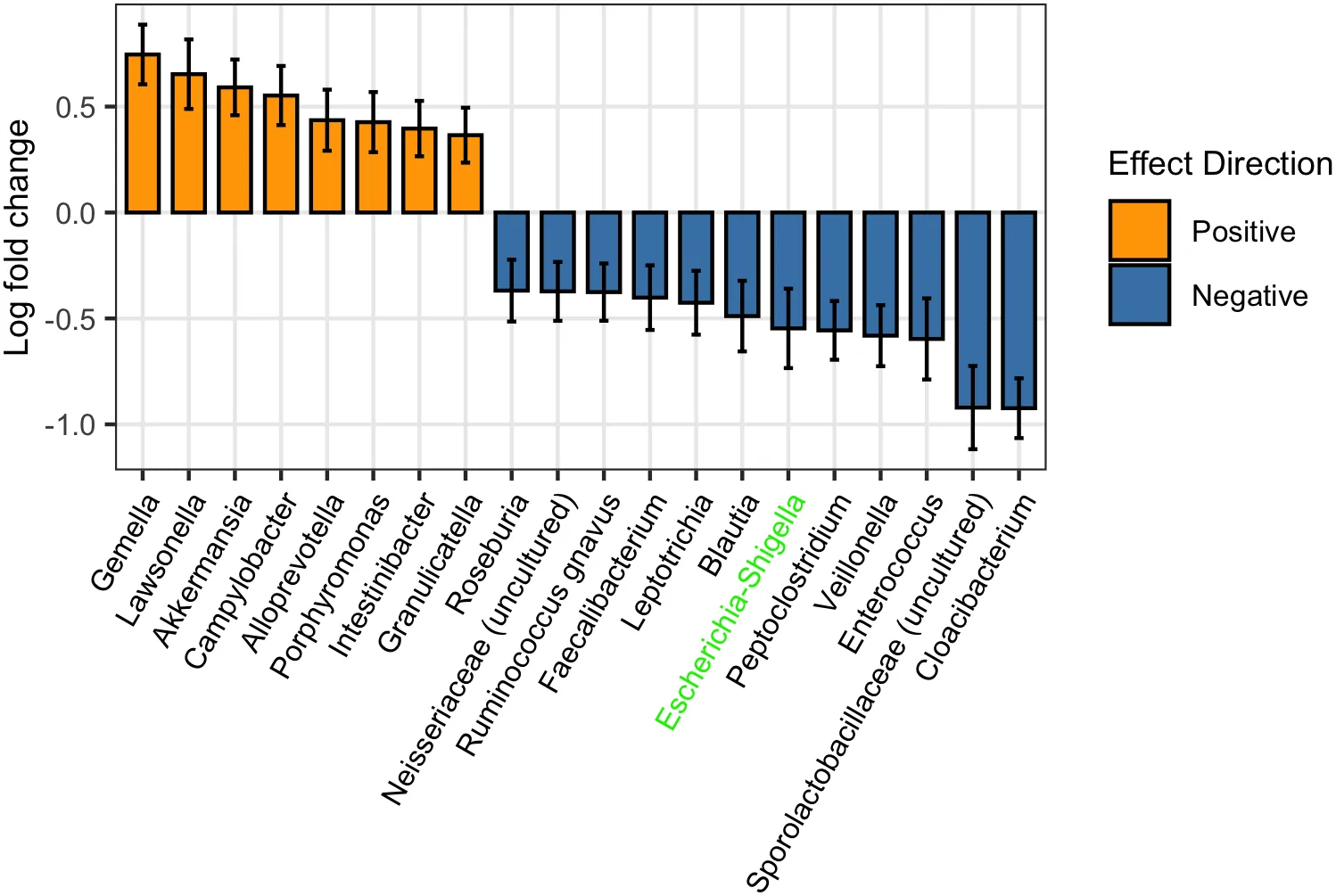
Specific bacterial genera are enriched in CRC tumor tissue based on MSI status. Differential abundance of taxa at the genera level using ANCOM-BC2, adjusted for age at diagnosis, sex, smoking history, and tumor location. Log-fold change in relative abundance plotted by the different genera that were statistically significant after false discovery rate correction. Genera that were enriched in MSI-high tumors are in orange and genera that were enriched in MSS/MSI-low tumors are blue. Genera labels highlighted in green were robust to sensitivity analysis of variation in pseudocounts.

Differential abundance analysis of 45 genera by MSI status with ANCOM-BC identified a set of 20 genera that were statistically significant after controlling FDR and adjusting for sex, age at diagnosis, smoking history, and tumor location. There were 8 genera that were enriched in MSI-high tumors: *Gemella, Lawsonella, Akkermansia, Campylobacter, Alloprevotella, Porphyromonas, Intestinibacter,* and *Granulicatella*, none of which was robust to sensitivity analysis varying the pseudocounts (Fig 3). A further 12 genera were enriched in MSS/MSI-low tumors, *Roseburia, Neisseriaceae, Ruminococcus gnavus, Faecalibacterium, Leptotrichia, Blautia, Escherichia-Shigella, Peptoclostridium, Veillonella, Enterococcus, Sporolactobacillaceae,* and *Cloacibacterium* (Fig 3). Of these, only *Escherichia-Shigella* was robust to pseudocount sensitivity analysis. To see how sensitive these results were to the level of taxonomic resolution, we performed ANCOM-BC at the family level and observed consistent enrichment of the family-level taxa containing the genera identified as differentially abundant. There was mixed representation of the families expected based on the genera level analysis; the families *Lawsonellaceae, Prevotellaceae, Clostridiaceae, Lachnospiraceae, Neisseriaceae,* and *Synergistota* were not differentially abundant as would be expected based on the genera-level analysis (S5 Fig). Interestingly, the family *Weeksellaceae* was depleted in MSI-high tumors though none of its members were significantly associated at the genera level (S5 Fig). Sex-specific differential abundances of genera by MSI status were observed as well (S6 Fig); where main analysis findings were replicated, enrichment by MSI status was restricted to one sex. In male participants, *Lawsonella* showed the strongest enrichment in MSI-high tumors while *Veillonella* showed the strongest depletion. Among female participants, *Cloacibacterium* was the most strongly depleted in MSI-high tumors while only *Thermicanus* was statistically significantly enriched. Differential abundance in *Anaerococcus*, *Brevundimonas*, *Faecalibacterium*, *Peptoclostridium,* and *Thermicanus* by MSI status showed inverse enrichment patterns between sexes (S6 Fig). Finally, we repeated ANCOM-BC at the genera level with only *F. nucleatum* specific ASVs included in the *Fusobacterium* genus; in this analysis, *Fusobacterium* was found to be both statistically significantly enriched in MSI-high tumors and robust to pseudocount sensitivity analysis (S7 Fig).

## Discussion

In this study, we explored the relationship between the MSI phenotype and CRC tumor microbiome alpha-diversity among CRC patients using an adaptive alpha-diversity measure that incorporates several potentially important features of community structure. We then searched for specific genera that were differentially abundant by MSI phenotype. Although none of the individual alpha-diversity measures nor the adaptive measure were statistically significantly associated with MSI status in the main analyses, participants with higher tumor alpha-diversity tended to have lower odds of MSI-high CRC across all individual alpha-diversity measures and in the adaptive measure. Furthermore, 20 genera were identified as differentially abundant in tumor tissue based on MSI status after adjustment for important confounding factors with patterns that differed by participant sex. *Fusobacterium* was found to be enriched in MSI-high tumors and robust to sensitivity analysis only after filtering *Fusobacterium* ASVs to those identified as *F. nucleatum* and its subspecies.

A number of previous studies have explored the relationship between MSI status and the specific members of the CRC tumor microbiome [8–11,17,71], but exploration of the relationship between alpha-diversity and MSI status has been limited [14,16]. It has been previously reported that tumor alpha-diversity is elevated in MSI-high tumors compared to MSS/MSI-low tumors across several alpha-diversity measures [14], whereas other work has reported no statistically significant associations between MSI status and tumor alpha-diversity in CRC tumors [16]. In contrast to these other works, our results are generally consistent with a null or inverse association between alpha-diversity and odds of MSI-high status in CRC, since the Shannon index and Simpson diversity tended towards an inverse association with MSI-high tumors on the ENS scale. There are a number of possible reasons for an observed lack of a strong association; for example, the *16S rRNA* gene sequencing was performed on FFPE tumor samples after a minimum of 12 years of storage which has been shown to decrease DNA quality and decrease alpha-diversity over time [72]. To our knowledge, our study is the first to examine the association between tumor alpha-diversity and MSI status using phylogeny-based measures of alpha-diversity, which may be important if greater phylogenetic distances between community taxa are strongly associated with MSI status. We found little evidence for a phylogeny-based effect, though we do see that prioritization of the most abundant taxa in a community tended to produce greater point estimates than abundance agnostic alpha-diversity measures (i.e., Richness, PD) using alpha-diversity measures on the ENS scale. This may indicate that the more abundant taxa are the drivers of any true association between alpha-diversity and MSI status. Furthermore, in sex-stratified aMiAD analysis, we saw stronger evidence of relationships between PE and PQE with MSI status in male participants, potentially indicating sex-specific relationships between alpha-diversity and MSI status, and supporting the importance of phylogenetic measures in understanding these relationships.

Transforming alpha-diversity measures to ENS values can provide useful information about how different features of community structure affect associations with alpha-diversity. Comparing measures of alpha-diversity is challenging because most measures are on different scales; thus, direct comparisons of their actual values are uninformative, and it is unclear to what degree differences in their strength of association are due to their scale versus prioritization of different community features (e.g., abundance). ENS values allow for the direct comparisons of alpha-diversity measures by transforming them to a common scale that can be framed as the number of equally common, equally distantly related taxa required to yield the same original value of the input alpha-diversity metric [63,73]. This becomes informative in interpreting strengths of association and direction; in our study we see that prioritization of the most abundant taxa in a community tended to strengthen the association between alpha-diversity and MSI status across both non-phylogenetic and phylogenetic alpha-diversity measures. The transformation to ENS values also made it clear that the Shannon index and Simpson index had similar estimates for their association with MSI status, which was not apparent in their original forms. Furthermore, ENS values are interpretable on a linear scale that lends itself to more naturalistic interpretations of effect estimates. Phylogeny-based alpha-diversity measures and ENS values are valuable tools for exploring how the microbiome is related to different disease features and understanding what microbial community features drive those relationships.

There have been efforts to characterize differentially abundant taxa in CRC tumors based on MSI status; Jin et al. [14] identified 17 genera that were differentially abundant by MSI status using *16S rRNA* gene sequencing data. The current study and Jin et al. find statistically significant enrichment of *Akkermansia* in MSI-high tumors compared to MSS/MSI-low tumors, although findings are in conflict with respect to other genera (e.g., *Faecalibacterium* and *Roseburia*). Notably, Jin et al. found enrichment of *Fusobacterium* in MSI-high tumors whereas, in our main analysis, it was not statistically significantly associated with MSI-status. Purcell et al. [17] found enrichment of *Fusobacterium hwasookii* and *Porphyromonas gingivalis* in tumors with gene expression profiles aligning with MSI-high tissues, supporting the finding of enrichment of *Porphyromonas* in MSI-high tumors from the current study. Patient sex has been observed to modify relationships between the gut microbiome and CRC lesions [70], but it has not been examined in the context of MSI status specifically; we found sex-specific differential abundance of bacterial genera by MSI status, with several of the main analysis findings being restricted to a single sex (e.g., *Lawsonella* and *Veillonella*) or following inverse enrichment patterns between sexes (e.g., *Thermicanus*). Other studies have performed much more targeted differential abundance analyses of microbes by MSI status [15,16], with a particular emphasis on *Fusobacterium* [10,11,71]. The lack of a strong association of *Fusobacterium* with MSI status in the current study’s ANCOM-BC analysis was likely a consequence of heterogeneity in the species represented in the ASVs classified as *Fusobacterium*: some of the *Fusobacterium* ASVs were classified as *Fusobacterium mortiferum* and *Fusobacterium necrophorum*, or lacked deeper resolution, so we re-ran ANCOM-BC at the genus level with only *Fusobacterium* ASVs that could be mapped to *F. nucleatum* or its subspecies. In this analysis, *Fusobacterium* were robustly enriched in the MSI-high tumors. Our analysis of effect modification of Shannon diversity on MSI status by *F. nucleatum* tumor positivity supports an association between *F. nucleatum* positivity and increased odds of MSI-high CRC, at least in the absence of interaction between diversity and *F. nucleatum* positivity.

This study has several key strengths. The inclusion of phylogenetic measures of alpha-diversity incorporated valuable information about the plausible drivers of any observed association. We were also able to adjust for important confounders of the relationship between alpha-diversity and MSI status, which have not been addressed in many previous works. This study also has important limitations. Samples used to characterize MSI status and alpha-diversity were collected at the same time, so we cannot address temporality of these associations nor the direction of any causal effects. The direction of causal effects is also obscured by the fact that we made no distinction between sporadic and inherited instances of MSI, and inherited MSI necessarily predates the formation of the colorectal microbiome. There may also be additional unmeasured confounding present in these results, from factors such as race (as a proxy for racialized disparities), alcohol consumption, and diet, that we were not able to adjust for in our analyses. Furthermore, while *16S rRNA* gene sequencing of the V4 hypervariable region is fast and affordable, the resolution of taxonomic classification is broadly limited to the genus level. As a result, the associations measured are aggregated across all species, subspecies, and strains, which may attenuate measured associations if sub-taxa act in opposing directions or have no effect.

## Conclusions

In this study, we explored the relationship between CRC tumor microbial diversity and MSI status. Although we did not find statistically significant evidence of an association between the set of alpha-diversity measures and MSI status, we did identify 20 differentially abundant genera by MSI status, with 8 genera enriched in MSI-high tumors and 12 being depleted in MSI-high tumors; these include several novel genera identified as differentially abundant. Future work should examine these genera for species that may have roles in the progression and etiology of CRC. Furthermore, the relationship between alpha-diversity and MSI status remains unclear, so future work is needed to elucidate this relationship.

## Supporting information

S1 TableSummary of the different alpha-diversity metrics and their formulation.Here, *S* is the set of all taxa in a sample, *p*_*i*_ and *p*_*j*_ are the proportion of taxa i in the sample, *l*_*i*_ is the phylogenetic distance between taxa i and the phylogeny root, *d*_*i,j*_ is the phylogenetic distance between taxa i and taxa j, and $\underset{―}{T}$ is the estimated height of the phylogeny. We assumed a standardized phylogeny with a height of 1 in analyses. Abbreviations: ENS, effective number of species.(TIFF)

S2 Table Summary of Shannon diversity across different participant characteristics by MSI status and overall. (TIFF)

S1 Fig Alpha-diversity measures on the effective number of species (ENS) scale reduces variation in effect estimates. Logistic regression results of alpha-diversity metrics on odds of having MSI-high CRC. Analysis adjusted for sex, age at diagnosis, smoking history, and tumor location. Models fit for both the nominal alpha-diversity metrics and on the ENS scale (here, ASVs). P-values for each estimate presented along each estimate. Note that Richness and PD are unchanged on the ENS scale as formulated here. Abbreviations: PD; Phylogenetic Diversity; PE, Phylogenetic Entropy; PQE, Phylogenetic Quadratic Entropy;ASVs, amplicon sequence variants.
(TIFF)

S2 Fig Adaptive and individual measures of alpha-diversity show inverse relationships with MSI status on effective number of species scale. Individual effect score estimates (MiDivES: light blue) from aMiAD analysis of the 6 alpha-diversity metrics transformed to effective number of species (ENS) values and the effect estimate of the adaptive measure aMiAD (aMiDvES: dark blue). Effect score estimates below 0 represent negative associations of alpha-diversity with MSI status, with the magnitude of the result indicating the relative strengths of the associations. Abbreviations: PD; Phylogenetic Diversity; PE, Phylogenetic Entropy; PQE, Phylogenetic Quadratic Entropy; MiDivES, Microbial Diversity Effect Score; aMiAD, adaptive microbiome alpha-diversity-based association analysis.
(TIFF)

S3 Fig Associations between individual measures of alpha-diversity and MSI status on effective number of species scale differ by sex. Individual effect score estimates from aMiAD analysis of the 6 alpha-diversity metrics transformed to effective number of species (ENS) values and the effect estimate of the adaptive measure aMiAD stratified by sex. Effect score estimates below 0 represent negative associations of alpha-diversity with MSI status, with the magnitude of the result indicating the relative strengths of the associations. Female participants (dark blue) and male participants (light blue) modeled separately. P-values shown for each estimate. Abbreviations: PD; Phylogenetic Diversity; PE, Phylogenetic Entropy; PQE, Phylogenetic Quadratic Entropy; MiDivES, Microbial Diversity Effect Score; aMiAD, adaptive microbiome alpha-diversity-based association analysis.
(TIFF)

S4 Fig Shannon diversity shows stronger association with MSI status in the *F. nucleatum* negative CRC tumor tissue. Logistic regression results of the estimated association of Shannon diversity on MSI status as odds ratios modelled on participant subsets with and without *F. nucleatum* positivity, adjusted for age at diagnosis, sex, smoking history, and tumor location. P-values of respective odds ratios shown.
(TIFF)

S5 Fig Specific bacterial families are enriched in CRC tumor tissue based on MSI status. Differential abundance of taxa at the family level using ANCOM-BC2, adjusted for age at diagnosis, sex, smoking history, and tumor location. Log-fold change in relative abundance plotted by the different families that were statistically significant after false discovery rate correction using the Benjamini-Hochberg method. Families that were enriched in MSI-high tumors are in orange and families that were enriched in MSS/MSI-low tumors are blue. Families labels highlighted in green were robust to sensitivity analysis of variation in pseudocounts.
(TIFF)

S6 Fig Sex-stratified ANCOM-BC2 shows sex specific associations between tumor genera and MSI status. Differential abundance of taxa at the genus level using ANCOM-BC2, adjusted for age at diagnosis, smoking history, and tumor location. Models were stratified by participant sex, with results from the female and male participant subsets in dark blue and light blue, respectively. Log-fold change in relative abundance plotted by the different genera that were statistically significant (asterisks) in at least one sex after false discovery rate correction using the Benjamini-Hochberg method. Genera that did not meet abundance threshold in one sex that were statistically significant in the other sex are also included.
(TIFF)

S7 Fig *Fusobacterium* is differentially abundant in CRC tumor tissue based on MSI status after enrichment for *F. nucleatum* specific ASVs. Differential abundance of taxa at the genus level using ANCOM-BC2, adjusted for age at diagnosis, sex, smoking history, and tumor location. *Fusobacterium* ASVs were subset to only ASVs that were identified as one of the *F. nucleatum* subspecies (*F. animalis*, *F. nucleatum*, *F. polymorphum*, and *F. vincentii*) by the top hit using BLASTn. Log-fold change in relative abundance plotted by the different genera that were statistically significant after false discovery rate correction with Benjamini-Hochberg method. Genera that were enriched in MSI-high tumors are in orange and genera that were enriched in MSS/MSI-low tumors are blue. Genera labels highlighted in green are robust to sensitivity analysis of variation in pseudocounts.
(TIFF)

S1 File Estimates of Shannon index and *F. nucleatum* positivity with MSI status. Estimated odds ratios (OR) for all variables in logistic regression models of Shannon index and *F. nucleatum* positivity on MSI status with and without interaction between the two measures (results from Fig 2), adjusted for age at diagnosis, sex, smoking history, sex, and tumor location.
(XLSX)
