# Supplementary material for: Tumor microbial biodiversity and microsatellite instability in colorectal cancer
Source: PLoS One. 2026 Sep 25;21(9):e0359555. doi: 10.1371/journal.pone.0359555 (PMC13614546; doi:10.1371/journal.pone.0359555)
Supplement: Supplementary file 1 [file pone.0359555.s012.docx]

August 6, 2026

RE: Response to reviewer comments for PLOS ONE submission PONE-D-26-16915

To Reviewer and members of the Editorial Board:

We would like to thank the editors and reviewers for the opportunity to revise and resubmit our paper, entitled “Tumor microbial biodiversity and microsatellite instability in colorectal cancer”. We deeply appreciate the valuable feedback that, we believe, has led to an improved paper. As described below, we have addressed the reviewers’ and editor’s comments and have revised the manuscript accordingly. Additional disclosure changes are detailed in the resubmission cover letter.

**Response to Editor comments regarding journal requirements:**

**1. Please ensure that your manuscript meets PLOS ONE's style requirements, including those for file naming.**

We have reviewed our manuscript file and made minor formatting changes to ensure that headings are formatted as headings and are of the required font size. All files for our revision are named as required per journal requirements.

**2. Please provide additional details regarding participant consent. In the ethics statement in the Methods and online submission information, please ensure that you have specified (1) whether consent was informed and (2) what type you obtained (for instance, written or verbal, and if verbal, how it was documented and witnessed). If your study included minors, state whether you obtained consent from parents or guardians. If the need for consent was waived by the ethics committee, please include this information.**

We have modified the text in the Methods section of our manuscript to more accurately reflect consenting protocols used in our parent study. Specifically, as now noted on lines 88-89 of the track-changed version of our manuscript revision, written informed consent was obtained from participants for the retrieval and use of tissue biospecimens and medical records, while verbal consent was obtained via trained interviewers for the collection and use of questionnaire-based information.

**3. Please provide an amended Funding Statement declaring commercial affiliations, as well as a statement regarding the Role of Funders in your study. If the funding organization did not play a role in the study design, data collection and analysis, decision to publish, or preparation of the manuscript and only provided financial support in the form of authors' salaries and/or research materials, please review your statements relating to the author contributions, and ensure you have specifically and accurately indicated the role(s) that these authors had in your study. You can update author roles in the Author Contributions section of the online submission form.**

**Please also include the following statement within your amended Funding Statement.**

**“The funder provided support in the form of salaries for authors [insert relevant initials], but did not have any additional role in the study design, data collection and analysis, decision to publish, or preparation of the manuscript. The specific roles of these authors are articulated in the ‘author contributions’ section.”**

**If your commercial affiliation did play a role in your study, please state and explain this role within your updated Funding Statement.**

**Please also provide an updated Competing Interests Statement declaring this commercial affiliation along with any other relevant declarations relating to employment, consultancy, patents, products in development, or marketed products, etc. Within your Competing Interests Statement, please confirm that this commercial affiliation does not alter your adherence to all PLOS ONE policies on sharing data and materials by including the following statement: "This does not alter our adherence to PLOS ONE policies on sharing data and materials.” If this adherence statement is not accurate and there are restrictions on sharing of data and/or materials, please state these.**

**Please include both an updated Funding Statement and Competing Interests Statement in your cover letter. We will change the online submission form on your behalf.**

We have included this information in our cover letter, stating clearly that no commercial entity played any role in the conduct of this work or preparation of our manuscript.

**4. We note that you have indicated that there are restrictions to data sharing for this study. PLOS only allows data to be available upon request if there are legal or ethical restrictions on sharing data publicly. For more information on unacceptable data access restrictions, please see** [**http://journals.plos.org/plosone/s/data-availability#loc-unacceptable-data-access-restrictions**](https://urldefense.com/v3/__http:/journals.plos.org/plosone/s/data-availability*loc-unacceptable-data-access-restrictions__;Iw!!K-Hz7m0Vt54!i0XlYpD9S0D9JLKvaAVDr47nThgvQ0Dr9PXxz6R5kcWaUxtXeb63hf1cPcpsKpO6itZbsLCl04NuxI-T$)**.**

**Before we proceed with your manuscript, please address the following prompts:**

**a) If there are ethical or legal restrictions on sharing a de-identified data set, please explain them in detail (e.g., data contain potentially identifying or sensitive patient information, data are owned by a third-party organization, etc.) and who has imposed them (e.g., a Research Ethics Committee or Institutional Review Board, etc.). Please also provide contact information for a data access committee, ethics committee, or other institutional body to which data requests may be sent.**

The Institutional Review Board for the Fred Hutchinson Cancer Center, which issues Institutional Certificates for the submission of de-identified data to public databases and journals, has restricted the study data that can be shared publicly. These restrictions are due to a lag in our inclusion of required language in our Informed Consents describing the possible sharing of study data to public databases. Consequently, the IRB at the Fred Hutchinson Cancer Center has explicitly excluded data that can be shared publicly based on the version and the date the consent was signed. We can, however, directly share all of the data generated for this manuscript via an application for collaboration to the Colon Cancer Family Registry (CCFR).

**Response to Reviewer Comments:**

*Please note that the line numbers in direct responses correspond to the tracked changes version of the revised manuscript.

**Reviewer 1:**

**This work is potentially interesting because CRC is one of the most common cancers in the world, or at least in developed countries. However, its analysis stratification fails to take into account the enormous gender differences recently reported in this area and, instead, considers sex a variable to be adjusted for, along with age at diagnosis, smoking habits, and other factors. The existence of substantial gender disparities in the composition of the gut microbiome has a huge impact on both the clinical and molecular characteristics of CRC. Therefore, failing to consider this aspect and not stratifying the data by gender risks "masking" important findings that could emerge from the analyses, and which could also suggest gender-specific diagnostic and therapeutic approaches.

Major point
Throughout the entire study, the authors never mention the gender differences observed in this type of tumor, which, precisely in terms of microsatellite instability, presents a significant difference between men and women, as is also evident from the data reported by the authors themselves in their sample description. Therefore, to be innovative and original, all aspects considered in the study should be reviewed by stratifying the analyses based on gender. This could yield interesting statistical significance.**

We agree with the reviewer that a person’s sex may modify relationships between the microbiome and microsatellite instability, and that a deeper investigation into this was warranted in our approach. In response, we have added additional sensitivity analyses (lines 230-233, 266-268, and 309-316 in the tracked-changes version), stratified on participant sex, for both the alpha-diversity models and the differential abundance analysis. We also include a brief discussion of these results (lines 359-362, and 391-395).

**Reviewer 2:**

**The only aspect that I believe represents a limitation of the study is the failure to assess sex-related differences. Given the availability of a large cohort and the inclusion of sex as a patient characteristic, it would have been appropriate to include stratified analyses or to evaluate possible interactions between sex, MSI status, and microbiome composition. Such an investigation could provide additional information of biological and clinical relevance, especially in light of evidence in the literature documenting differences in incidence, molecular characteristics, and prognosis of colorectal cancer between men and women. The inclusion of these analyses would further strengthen the value and originality of the study.**

As described above, in response to Reviewer 1’s very similar comment, we agree with Reviewer 2 that the inclusion of additional analyses examining sex-based effect modification of the MSI status and microbiome relationship would add depth and originality to our study. We again appreciate this suggestion. We have updated the manuscript with supplemental sex-stratified analyses (lines 230-233, 266-268, and 309-316) and a discussion of these results (lines 359-362, and 391-395).
