# Supplementary material for: Tumor microbial biodiversity and microsatellite instability in colorectal cancer
Source: PLoS One. 2026 Sep 25;21(9):e0359555. doi: 10.1371/journal.pone.0359555 (PMC13614546; doi:10.1371/journal.pone.0359555)
Supplement: Peer review history

# Peer review: Decision Letter 0

**DOI:** 10.1371/journal.pone.0359555.r001

**Authors:** David Ojcius

9 Jul 2026

Dear Dr. Phipps,

Thank you for submitting your manuscript to PLOS ONE. After careful consideration, we feel that it has merit but does not fully meet PLOS ONE’s publication criteria as it currently stands. Therefore, we invite you to submit a revised version of the manuscript that addresses the points raised during the review process. In particular, the reviewers request that the manuscript take into account gender differences.

Please submit your revised manuscript by Aug 23 2026 11:59PM. If you will need more time than this to complete your revisions, please reply to this message or contact the journal office at plosone@plos.org. When you're ready to submit your revision, log on to https://www.editorialmanager.com/pone/ and select the 'Submissions Needing Revision' folder to locate your manuscript file.

- A letter that responds to each point raised by the academic editor and reviewer(s). You should upload this letter as a separate file labeled 'Response to Reviewers'.
- A marked-up copy of your manuscript that highlights changes made to the original version. You should upload this as a separate file labeled 'Revised Manuscript with Track Changes'.
- An unmarked version of your revised paper without tracked changes. You should upload this as a separate file labeled 'Manuscript'.

If you would like to make changes to your financial disclosure, please include your updated statement in your cover letter. Guidelines for resubmitting your figure files are available below the reviewer comments at the end of this letter.

If applicable, we recommend that you deposit your laboratory protocols in protocols.io to enhance the reproducibility of your results. Protocols.io assigns your protocol its own identifier (DOI) so that it can be cited independently in the future. For instructions see: https://journals.plos.org/plosone/s/submission-guidelines#loc-laboratory-protocols. Additionally, PLOS ONE offers an option for publishing peer-reviewed Lab Protocol articles, which describe protocols hosted on protocols.io. Read more information on sharing protocols at https://plos.org/protocols?utm_medium=editorial-email&utm_source=authorletters&utm_campaign=protocols.

As the corresponding author, your ORCID iD is verified in the submission system and will appear in the published article. PLOS supports the use of ORCID, and we encourage all coauthors to register for an ORCID iD and use it as well. Please encourage your coauthors to verify their ORCID iD within the submission system before final acceptance, as unverified ORCID iDs will not appear in the published article. *Only*  the individual author can complete the verification step; PLOS staff *cannot*  verify ORCID iDs on behalf of authors.

We look forward to receiving your revised manuscript.

Kind regards,

David M. Ojcius

Academic Editor

PLOS One

Journal Requirements:

When submitting your revision, we need you to address these additional requirements.

1.Please ensure that your manuscript meets PLOS ONE's style requirements, including those for file naming. The PLOS ONE style templates can be found at

https://journals.plos.org/plosone/s/file?id=wjVg/PLOSOne_formatting_sample_main_body.pdf and

https://journals.plos.org/plosone/s/file?id=ba62/PLOSOne_formatting_sample_title_authors_affiliations.pdf

2. Please provide additional details regarding participant consent. In the ethics statement in the Methods and online submission information, please ensure that you have specified (1) whether consent was informed and (2) what type you obtained (for instance, written or verbal, and if verbal, how it was documented and witnessed). If your study included minors, state whether you obtained consent from parents or guardians. If the need for consent was waived by the ethics committee, please include this information.

If you are reporting a retrospective study of medical records or archived samples, please ensure that you have discussed whether all data were fully anonymized before you accessed them and/or whether the IRB or ethics committee waived the requirement for informed consent. If patients provided informed written consent to have data from their medical records used in research, please include this information.

3. Thank you for stating the following in the Competing Interests section:

“I have read the journal's policy and the authors of this manuscript have the following competing interests:

Calen Mendall performed contracted research with Veterans affairs during the period when this work was conducted, unrelated to the present study.

Claire E. Thomas was an epidemiology contractor with Pfizer during the period when this work was conducted, unrelated to the present study. She is currently an employee of PreOncology, unrelated to the present study.

Shuji Ogino served as a consultant for Sanofi Pasteur S.A., unrelated to the present study.”

We note that one or more of the authors are employed by a commercial company: Pfizer, PreOncology and Sanofi Pasteur S.A

1. Please provide an amended Funding Statement declaring this commercial affiliation, as well as a statement regarding the Role of Funders in your study. If the funding organization did not play a role in the study design, data collection and analysis, decision to publish, or preparation of the manuscript and only provided financial support in the form of authors' salaries and/or research materials, please review your statements relating to the author contributions, and ensure you have specifically and accurately indicated the role(s) that these authors had in your study. You can update author roles in the Author Contributions section of the online submission form.

Please also include the following statement within your amended Funding Statement.

“The funder provided support in the form of salaries for authors [insert relevant initials], but did not have any additional role in the study design, data collection and analysis, decision to publish, or preparation of the manuscript. The specific roles of these authors are articulated in the ‘author contributions’ section.”

If your commercial affiliation did play a role in your study, please state and explain this role within your updated Funding Statement.

2. Please also provide an updated Competing Interests Statement declaring this commercial affiliation along with any other relevant declarations relating to employment, consultancy, patents, products in development, or marketed products, etc.

Within your Competing Interests Statement, please confirm that this commercial affiliation does not alter your adherence to all PLOS ONE policies on sharing data and materials by including the following statement: "This does not alter our adherence to  PLOS ONE policies on sharing data and materials.” (as detailed online in our guide for authors http://journals.plos.org/plosone/s/competing-interests) . If this adherence statement is not accurate and  there are restrictions on sharing of data and/or materials, please state these. Please note that we cannot proceed with consideration of your article until this information has been declared.

Please include both an updated Funding Statement and Competing Interests Statement in your cover letter. We will change the online submission form on your behalf.

4. We note that you have indicated that there are restrictions to data sharing for this study. PLOS only allows data to be available upon request if there are legal or ethical restrictions on sharing data publicly. For more information on unacceptable data access restrictions, please see http://journals.plos.org/plosone/s/data-availability#loc-unacceptable-data-access-restrictions.

Before we proceed with your manuscript, please address the following prompts:

a) If there are ethical or legal restrictions on sharing a de-identified data set, please explain them in detail (e.g., data contain potentially identifying or sensitive patient information, data are owned by a third-party organization, etc.) and who has imposed them (e.g., a Research Ethics Committee or Institutional Review Board, etc.). Please also provide contact information for a data access committee, ethics committee, or other institutional body to which data requests may be sent.

b) If there are no restrictions, please upload the minimal anonymized data set necessary to replicate your study findings to a stable, public repository and provide us with the relevant URLs, DOIs, or accession numbers. For a list of recommended repositories, please see

https://journals.plos.org/plosone/s/recommended-repositories. You also have the option of uploading the data as Supporting Information files, but we would recommend depositing data directly to a data repository if possible.

We will update your Data Availability statement on your behalf to reflect the information you provide.

5. If the reviewer comments include a recommendation to cite specific previously published works, please review and evaluate these publications to determine whether they are relevant and should be cited. There is no requirement to cite these works unless the editor has indicated otherwise.

[Note: HTML markup is below. Please do not edit.]

Reviewers' comments:

Reviewer's Responses to Questions

**Comments to the Author**

1. Is the manuscript technically sound, and do the data support the conclusions?

Reviewer #1: Yes

Reviewer #2: Yes

**********

2. Has the statistical analysis been performed appropriately and rigorously?

Reviewer #1: I Don't Know

Reviewer #2: Yes

**********

3. Have the authors made all data underlying the findings in their manuscript fully available?

The PLOS Data policy

Reviewer #1: Yes

Reviewer #2: Yes

**********

4. Is the manuscript presented in an intelligible fashion and written in standard English?

Reviewer #1: Yes

Reviewer #2: Yes

**********

Reviewer #1: The paper entitled “Tumor microbial biodiversity and microsatellite instability in colorectal cancer” by Calen Mendall, Meredith A.J. Hullar, Keith R. Curtis, Courtney M. Hill, Claire E. Thomas, Ningxin

Ma, Timothy W. Randolph, Rachel C. Malen, Adriana M. Reedy, Scott LaBrie2, John D. Potter, Shuji Ogino, Polly A. Newcomb, and Amanda I. Phipps examines the possible correlation between tumor microbiota biodiversity and the presence or absence of microsatellite instability. The analyses suggest a positive association, which, however, does not reach statistical significance, between MSI-high and low biodiversity, identifying 8 enriched genera and 12 depleted genera in tumors with high microsatellite instability. The analyses suggest a positive association, though not statistically significant, between MSI-high and low biodiversity, identifying 8 enriched and 12 depleted genera in tumors with high microsatellite instability.

In particular, the genera most enriched in MSI-high tumors were Gemella and Lawsonella, while Sporolactobacillaceae and Cloacibacterium were the most strongly depleted. Furthermore, Fusobacterium nucleatum was enriched in MSI-high tumors.

General comments

This work is potentially interesting because CRC is one of the most common cancers in the world, or at least in developed countries. However, its analysis stratification fails to take into account the enormous gender differences recently reported in this area and, instead, considers sex a variable to be adjusted for, along with age at diagnosis, smoking habits, and other factors.

The existence of substantial gender disparities in the composition of the gut microbiome has a huge impact on both the clinical and molecular characteristics of CRC. Therefore, failing to consider this aspect and not stratifying the data by gender risks "masking" important findings that could emerge from the analyses, and which could also suggest gender-specific diagnostic and therapeutic approaches.

Major point

Throughout the entire study, the authors never mention the gender differences observed in this type of tumor, which, precisely in terms of microsatellite instability, presents a significant difference between men and women, as is also evident from the data reported by the authors themselves in their sample description.

Therefore, to be innovative and original, all aspects considered in the study should be reviewed by stratifying the analyses based on gender. This could yield interesting statistical significance.

Reviewer #2: The manuscript addresses a topic of growing interest—namely, the relationship between the tumor microbiome and microsatellite instability (MSI) in colorectal cancer—using a large cohort and appropriate analytical methods. The study has several strengths, including the sample size, the use of advanced statistical approaches for analyzing microbial biodiversity and differential abundance, and the identification of potential associations between specific bacterial taxa and MSI status. Although some clarifications and further analysis are needed to strengthen the robustness of the conclusions, the manuscript is overall well-written, well-structured, and of potential interest to the journal’s readers. The only aspect that I believe represents a limitation of the study is the failure to assess sex-related differences. Given the availability of a large cohort and the inclusion of sex as a patient characteristic, it would have been appropriate to include stratified analyses or to evaluate possible interactions between sex, MSI status, and microbiome composition. Such an investigation could provide additional information of biological and clinical relevance, especially in light of evidence in the literature documenting differences in incidence, molecular characteristics, and prognosis of colorectal cancer between men and women. The inclusion of these analyses would further strengthen the value and originality of the study.

**********

what does this mean?). If published, this will include your full peer review and any attached files.

If you choose “no”, your identity will remain anonymous but your review may still be made public.

**Do you want your identity to be public for this peer review?**  For information about this choice, including consent withdrawal, please see our Privacy Policy

Reviewer #1: No

Reviewer #2: No

**********

[NOTE: If reviewer comments were submitted as an attachment file, they will be attached to this email and accessible via the submission site. Please log into your account, locate the manuscript record, and check for the action link "View Attachments". If this link does not appear, there are no attachment files.]

To ensure your figures meet our technical requirements, please review our figure guidelines: https://journals.plos.org/plosone/s/figures

You may also use PLOS’s free figure tool, NAAS, to help you prepare publication quality figures: https://journals.plos.org/plosone/s/figures#loc-tools-for-figure-preparation.

NAAS will assess whether your figures meet our technical requirements by comparing each figure against our figure specifications.

# Author comment: Author response to Decision Letter 1

**DOI:** 10.1371/journal.pone.0359555.r002

1 Sep 2026

Detailed responses to reviewer and editor comments are provided in the Response to Reviewer document. We have also pasted these comments and responses below.

To Reviewer and members of the Editorial Board:

We would like to thank the editors and reviewers for the opportunity to revise and resubmit our paper, entitled “Tumor microbial biodiversity and microsatellite instability in colorectal cancer”. We deeply appreciate the valuable feedback that, we believe, has led to an improved paper. As described below, we have addressed the reviewers’ and editor’s comments and have revised the manuscript accordingly. Additional disclosure changes are detailed in the resubmission cover letter.

Response to Editor comments regarding journal requirements:

1. Please ensure that your manuscript meets PLOS ONE's style requirements, including those for file naming.

We have reviewed our manuscript file and made minor formatting changes to ensure that headings are formatted as headings and are of the required font size. All files for our revision are named as required per journal requirements.

2. Please provide additional details regarding participant consent. In the ethics statement in the Methods and online submission information, please ensure that you have specified (1) whether consent was informed and (2) what type you obtained (for instance, written or verbal, and if verbal, how it was documented and witnessed). If your study included minors, state whether you obtained consent from parents or guardians. If the need for consent was waived by the ethics committee, please include this information.

We have modified the text in the Methods section of our manuscript to more accurately reflect consenting protocols used in our parent study. Specifically, as now noted on lines 88-89 of the track-changed version of our manuscript revision, written informed consent was obtained from participants for the retrieval and use of tissue biospecimens and medical records, while verbal consent was obtained via trained interviewers for the collection and use of questionnaire-based information.

3. Please provide an amended Funding Statement declaring commercial affiliations, as well as a statement regarding the Role of Funders in your study. If the funding organization did not play a role in the study design, data collection and analysis, decision to publish, or preparation of the manuscript and only provided financial support in the form of authors' salaries and/or research materials, please review your statements relating to the author contributions, and ensure you have specifically and accurately indicated the role(s) that these authors had in your study. You can update author roles in the Author Contributions section of the online submission form.

Please also include the following statement within your amended Funding Statement.

“The funder provided support in the form of salaries for authors [insert relevant initials], but did not have any additional role in the study design, data collection and analysis, decision to publish, or preparation of the manuscript. The specific roles of these authors are articulated in the ‘author contributions’ section.”

If your commercial affiliation did play a role in your study, please state and explain this role within your updated Funding Statement.

Please also provide an updated Competing Interests Statement declaring this commercial affiliation along with any other relevant declarations relating to employment, consultancy, patents, products in development, or marketed products, etc. Within your Competing Interests Statement, please confirm that this commercial affiliation does not alter your adherence to all PLOS ONE policies on sharing data and materials by including the following statement: "This does not alter our adherence to PLOS ONE policies on sharing data and materials.” If this adherence statement is not accurate and there are restrictions on sharing of data and/or materials, please state these.

Please include both an updated Funding Statement and Competing Interests Statement in your cover letter. We will change the online submission form on your behalf.

We have included this information in our cover letter, stating clearly that no commercial entity played any role in the conduct of this work or preparation of our manuscript.

4. We note that you have indicated that there are restrictions to data sharing for this study. PLOS only allows data to be available upon request if there are legal or ethical restrictions on sharing data publicly. For more information on unacceptable data access restrictions, please see http://journals.plos.org/plosone/s/data-availability#loc-unacceptable-data-access-restrictions.

Before we proceed with your manuscript, please address the following prompts:

a) If there are ethical or legal restrictions on sharing a de-identified data set, please explain them in detail (e.g., data contain potentially identifying or sensitive patient information, data are owned by a third-party organization, etc.) and who has imposed them (e.g., a Research Ethics Committee or Institutional Review Board, etc.). Please also provide contact information for a data access committee, ethics committee, or other institutional body to which data requests may be sent.

The Institutional Review Board for the Fred Hutchinson Cancer Center, which issues Institutional Certificates for the submission of de-identified data to public databases and journals, has restricted the study data that can be shared publicly. These restrictions are due to a lag in our inclusion of required language in our Informed Consents describing the possible sharing of study data to public databases. Consequently, the IRB at the Fred Hutchinson Cancer Center has explicitly excluded data that can be shared publicly based on the version and the date the consent was signed. We can, however, directly share all of the data generated for this manuscript via an application for collaboration to the Colon Cancer Family Registry (CCFR).

Response to Reviewer Comments:

*Please note that the line numbers in direct responses correspond to the tracked changes version of the revised manuscript.

Reviewer 1:

This work is potentially interesting because CRC is one of the most common cancers in the world, or at least in developed countries. However, its analysis stratification fails to take into account the enormous gender differences recently reported in this area and, instead, considers sex a variable to be adjusted for, along with age at diagnosis, smoking habits, and other factors. The existence of substantial gender disparities in the composition of the gut microbiome has a huge impact on both the clinical and molecular characteristics of CRC. Therefore, failing to consider this aspect and not stratifying the data by gender risks "masking" important findings that could emerge from the analyses, and which could also suggest gender-specific diagnostic and therapeutic approaches.

Major point

Throughout the entire study, the authors never mention the gender differences observed in this type of tumor, which, precisely in terms of microsatellite instability, presents a significant difference between men and women, as is also evident from the data reported by the authors themselves in their sample description. Therefore, to be innovative and original, all aspects considered in the study should be reviewed by stratifying the analyses based on gender. This could yield interesting statistical significance.

We agree with the reviewer that a person’s sex may modify relationships between the microbiome and microsatellite instability, and that a deeper investigation into this was warranted in our approach. In response, we have added additional sensitivity analyses (lines 230-233, 266-268, and 309-316 in the tracked-changes version), stratified on participant sex, for both the alpha-diversity models and the differential abundance analysis. We also include a brief discussion of these results (lines 359-362, and 391-395).

Reviewer 2:

The only aspect that I believe represents a limitation of the study is the failure to assess sex-related differences. Given the availability of a large cohort and the inclusion of sex as a patient characteristic, it would have been appropriate to include stratified analyses or to evaluate possible interactions between sex, MSI status, and microbiome composition. Such an investigation could provide additional information of biological and clinical relevance, especially in light of evidence in the literature documenting differences in incidence, molecular characteristics, and prognosis of colorectal cancer between men and women. The inclusion of these analyses would further strengthen the value and originality of the study.

As described above, in response to Reviewer 1’s very similar comment, we agree with Reviewer 2 that the inclusion of additional analyses examining sex-based effect modification of the MSI status and microbiome relationship would add depth and originality to our study. We again appreciate this suggestion. We have updated the manuscript with supplemental sex-stratified analyses (lines 230-233, 266-268, and 309-316) and a discussion of these results (lines 359-362, and 391-395).

Attachment Submitted filename: Response To Reviewers.docx

# Peer review: Decision Letter 1

**DOI:** 10.1371/journal.pone.0359555.r003

**Authors:** David Ojcius

15 Sep 2026

Tumor microbial biodiversity and microsatellite instability in colorectal cancer

PONE-D-26-16915R1

Dear Dr. Phipps,

We’re pleased to inform you that your manuscript has been judged scientifically suitable for publication and will be formally accepted for publication once it meets all outstanding technical requirements.

Within one week, you’ll receive an e-mail detailing the required amendments. When these have been addressed, you’ll receive a formal acceptance letter and your manuscript will be scheduled for publication.

An invoice will be generated when your article is formally accepted. Please note, if your institution has a publishing partnership with PLOS and your article meets the relevant criteria, all or part of your publication costs will be covered. Please make sure your user information is up-to-date by logging into Editorial Manager at Editorial Manager® and clicking the ‘Update My Information' link at the top of the page. For questions related to billing, please contact billing support.

If your institution or institutions have a press office, please notify them about your upcoming paper to help maximize its impact. If they’ll be preparing press materials, please inform our press team as soon as possible -- no later than 48 hours after receiving the formal acceptance. Your manuscript will remain under strict press embargo until 2 pm Eastern Time on the date of publication. For more information, please contact onepress@plos.org.

Kind regards,

David M. Ojcius

Academic Editor

PLOS One

Reviewers' comments:

Reviewer's Responses to Questions

**Comments to the Author**

Reviewer #1: All comments have been addressed

**********

2. Is the manuscript technically sound, and do the data support the conclusions?

Reviewer #1: Yes

**********

3. Has the statistical analysis been performed appropriately and rigorously?

Reviewer #1: Yes

**********

4. Have the authors made all data underlying the findings in their manuscript fully available?

The PLOS Data policy

Reviewer #1: Yes

**********

5. Is the manuscript presented in an intelligible fashion and written in standard English?

Reviewer #1: Yes

**********

Reviewer #1: All comments have been satisfactorily addressed. The authors might consider specifying in the title that they conducted a sex-specific analysis, to highlight the originality of their findings.

**********

what does this mean?). If published, this will include your full peer review and any attached files.

If you choose “no”, your identity will remain anonymous but your review may still be made public.

**Do you want your identity to be public for this peer review?**  For information about this choice, including consent withdrawal, please see our Privacy Policy

Reviewer #1: No

**********

# Editor report: Acceptance letter

**DOI:** 10.1371/journal.pone.0359555.r004

**Authors:** David Ojcius

PONE-D-26-16915R1

PLOS One

Dear Dr. Phipps,

I'm pleased to inform you that your manuscript has been deemed suitable for publication in PLOS One. Congratulations! Your manuscript is now being handed over to our production team.

At this stage, our production department will prepare your paper for publication. This includes ensuring the following:

* All references, tables, and figures are properly cited

* All relevant supporting information is included in the manuscript submission,

* There are no issues that prevent the paper from being properly typeset

You will receive further instructions from the production team, including instructions on how to review your proof when it is ready. Please keep in mind that we are working through a large volume of accepted articles, so please give us a few days to review your paper and let you know the next and final steps.

Lastly, if your institution or institutions have a press office, please let them know about your upcoming paper now to help maximize its impact. If they'll be preparing press materials, please inform our press team within the next 48 hours. Your manuscript will remain under strict press embargo until 2 pm Eastern Time on the date of publication. For more information, please contact onepress@plos.org.

You will receive an invoice from PLOS for your publication fee after your manuscript has reached the completed accept phase. If you receive an email requesting payment before acceptance or for any other service, this may be a phishing scheme. Learn how to identify phishing emails and protect your accounts at https://explore.plos.org/phishing.

If we can help with anything else, please email us at customercare@plos.org.

Thank you for submitting your work to PLOS One and supporting open access.

Kind regards,

PLOS One Editorial Office Staff

on behalf of

Dr. David M. Ojcius

Academic Editor

PLOS One
